# The bovine QTL viewer: a web accessible database of bovine Quantitative Trait Loci

**DOI:** 10.1186/1471-2105-7-283

**Published:** 2006-06-05

**Authors:** Pavana Polineni, Prathyusha Aragonda, Suresh R Xavier, Richard Furuta, David L Adelson

**Affiliations:** 1 Dept. of Animal Science, Texas A&M University, Mail Stop 2471, College Station, TX, 77843-2471, USA; 2 Dept. of Computer Science, Texas A&M University, Mail Stop 3112, College Station, TX, 77843-3112, USA

## Abstract

**Background:**

Many important agricultural traits such as weight gain, milk fat content and intramuscular fat (marbling) in cattle are quantitative traits. Most of the information on these traits has not previously been integrated into a genomic context. Without such integration application of these data to agricultural enterprises will remain slow and inefficient. Our goal was to populate a genomic database with data mined from the bovine quantitative trait literature and to make these data available in a genomic context to researchers via a user friendly query interface.

**Description:**

The QTL (Quantitative Trait Locus) data and related information for bovine QTL are gathered from published work and from existing databases. An integrated database schema was designed and the database (MySQL) populated with the gathered data. The bovine QTL Viewer was developed for the integration of QTL data available for cattle. The tool consists of an integrated database of bovine QTL and the QTL viewer to display QTL and their chromosomal position.

**Conclusion:**

We present a web accessible, integrated database of bovine (dairy and beef cattle) QTL for use by animal geneticists. The viewer and database are of general applicability to any livestock species for which there are public QTL data. The viewer can be accessed at .

## Background

Many important agricultural traits such as weight gain, milk fat content and intramuscular fat (marbling) in cattle are quantitative traits. While significant information regarding the mode of inheritance of these traits is available, most of this information is not integrated into a genomic context. As large amounts of genomic sequence data become available (estimated completion time of the bovine genome sequence is early 2006), they require livestock genome researchers to integrate sequence data not only with existing gene maps, but more importantly with Quantitative Trait Locus (QTL) and phenotype data. Without integration, application of these data to agricultural enterprise productivity will remain slow and inefficient. In order to facilitate this overall integration, there is a requirement for an integrated database of QTL of cattle and an analytical tool for this database such as a visualization component.

While there are livestock genetic map viewers [1,2] and at least one QTL database [3], to the best of our knowledge there is only one other bovine QTL viewer available [4], but it is not dynamic and is restricted to one meta-analysis of dairy QTL data [5]. Our inspiration for this effort was RatMap [6], but our code and database schema differ substantially from theirs. We have created a web accessible, integrated database of bovine QTL that will be extended to link to genome sequence data as it becomes available. Our QTL viewer is able to show the contents of the database in a manner suitable for both novice and expert users.

## Construction and content

**Figure 1:**
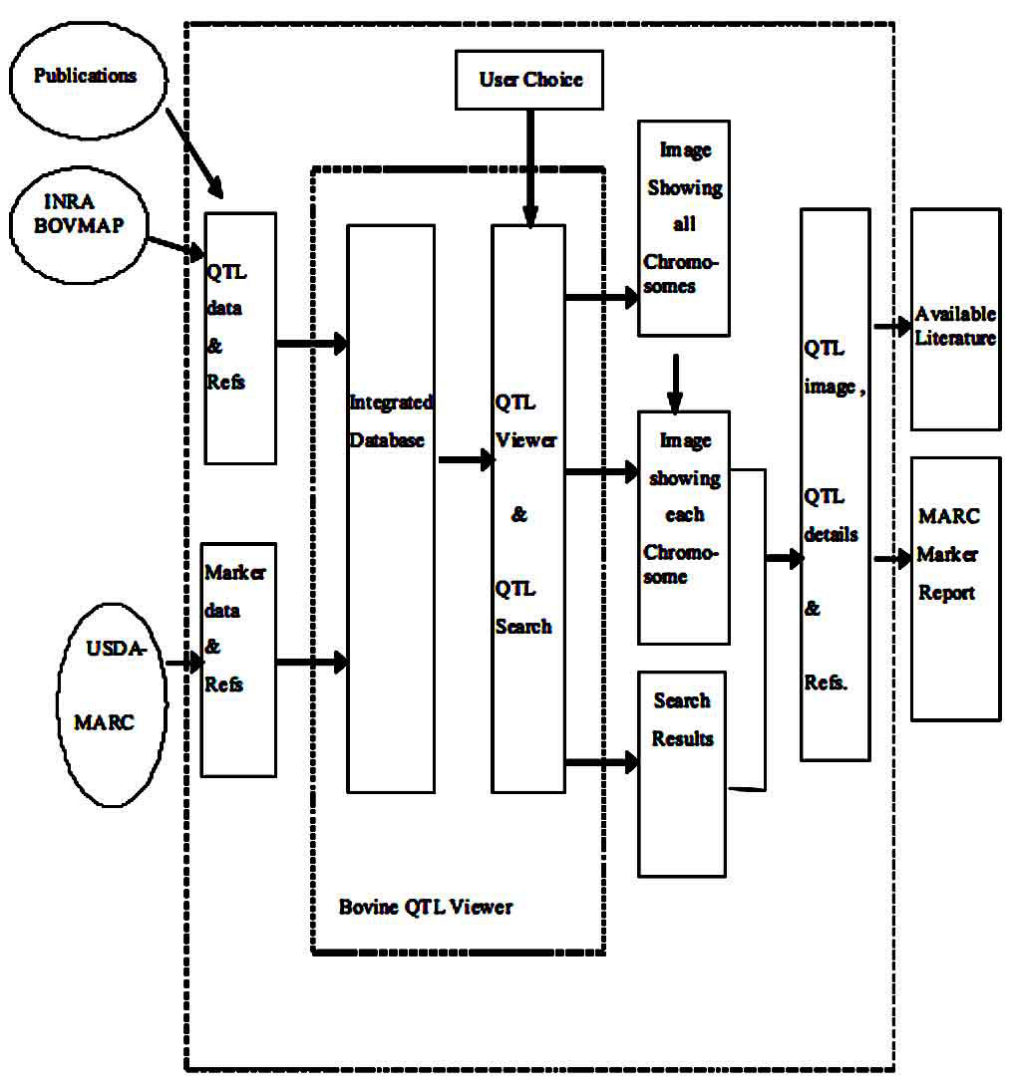
QTL viewer description. This is a diagrammatic view describing the inputs and outputs from the database and viewer. QTL and marker information are assembled from the literature or web sites and entered into the linked, discrete marker and QTL databases. The integrated marker/QTL database serves as the back end for queries emanating from the user interface (viewer). The viewer will display different views depending on user choices which can ultimately be resolved to single QTL details with links to literature and markers.

The QTL data and related information for bovine QTL are gathered from published work and from existing databases such as INRA BOVMAP [3] and USDA-MARC[1]. We elected to use the USDA-MARC map as our reference map and to display all QTL on the MARC map. This was done as a matter of convenience, as the MARC map is the most comprehensive bovine linkage map available. Every QTL mapping experiment results in the generation of a new map within which the QTL are localized. In order to represent QTL from different experiments on a single map, we needed to use a map that incorporated the markers used to define QTL. The MARC map fulfilled this criterion. In a small number of cases, markers have been used to map QTL and are not present on the MARC map. In these cases, the closest USDA marker that flanked the QTL marker were used as a proxy. An integrated database schema was designed and the database (MySQL) populated with the gathered data. Quarterly literature searches will allow frequent updating of the database. The bovine QTL Viewer was developed for the integration of QTL data available for cattle. The tool consists of an integrated database of bovine QTL and the QTL viewer to display QTL and their chromosomal position. As shown in Fig. 1, the database mainly consists of QTL data and Marker data.

**Figure 2:**
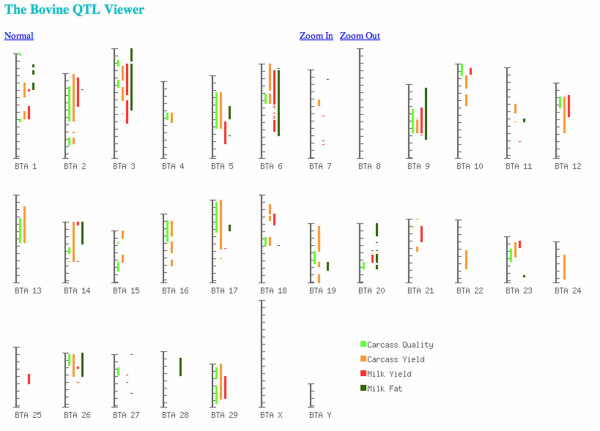
Genome view of multiple QTL. This screen shot shows the result of a query for QTL for carcass quality, carcass yield, milk yield and milk fat traits. The QTL regions are color coded and for ease of viewing multiple, overlapping QTL for any single trait are collapsed into a single color coded line. By selecting a single QTL line with the mouse, the user is able to transition to the single chromosome view, where all individual QTL are displayed (see Figure 3).

The database is independent of the viewer, which dynamically displays the query results (using the USDA MARC map as a the reference map) as graphical representations of chromosomes highlighted in the QTL regions (Fig. 2). Because the database is independent from the viewer it is a simple matter to add databases for other species making this a more versatile tool. The interface was designed after initially canvassing potential users and two rounds of feedback to ensure user desired features were incorporated into the design.

**Figure 3:**
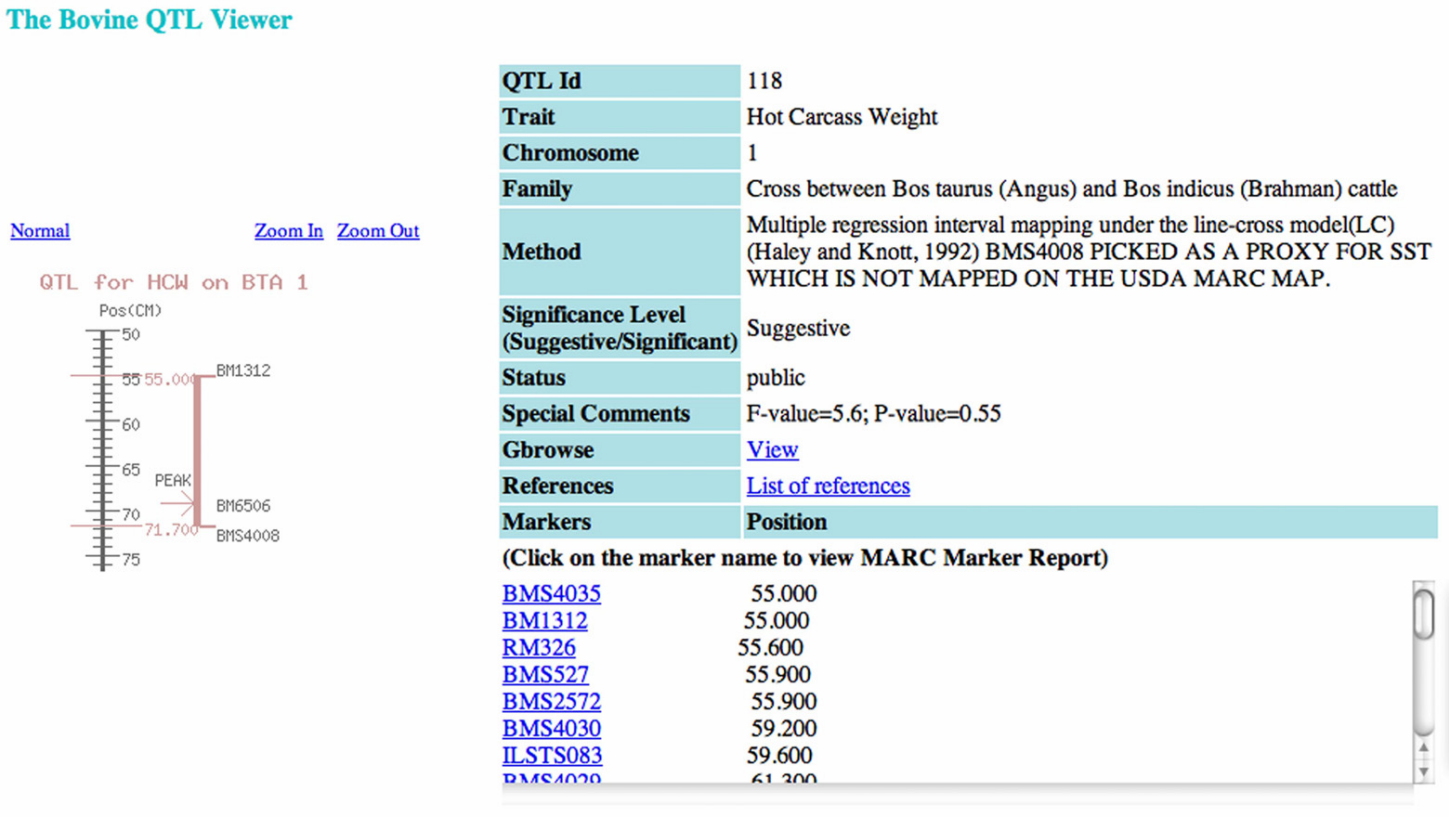
Detailed view of QTL. The critical interval of the QTL is shown along with peak marker location. QTL summary data such as QTL ID, statistical signficance, family structure and statistical methods are presented. Links to the USDA marker database are provided, along with links to references used. When a fully functional genome browser becomes available a link to that site will be provided here.

The user can then select a particular chromosome to view in detail by clicking on it. This chromosome view provides a detailed view of all QTL reported in the genome view generated previously. The user can go directly to the chromosome view by selecting a single chromosome at the time of the initial search. Individual QTL can then be viewed in detail (Fig. 3) and at this point the user can determine statistical significance, family structure, literature references and markers included within the QTL region. A future link out to a genome browser will be available from this QTL detail view.

**Figure 4:**
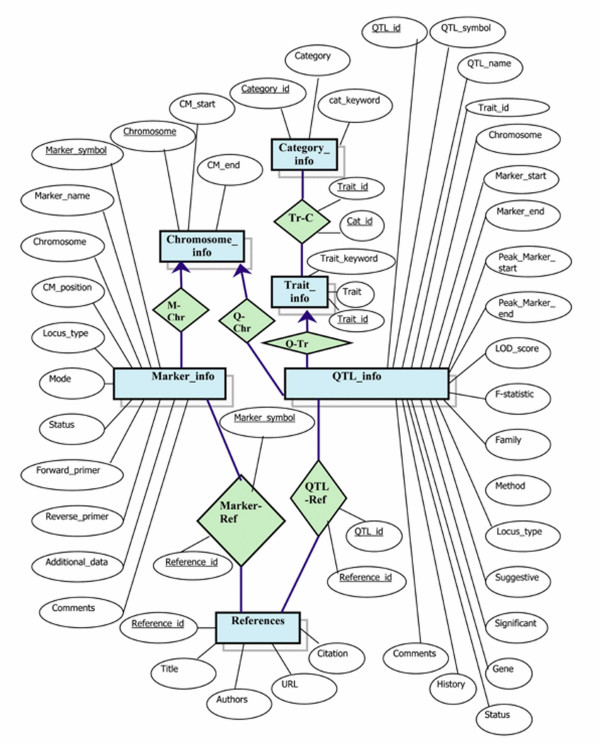
Entity-Relationship diagram of the integrated database. The integrated database consists of two major, linked divisions, the marker database, which is independent from the QTL database. This allows easy updating of either component of the database or potentially allowing the QTL database to be linked to multiple maps. QTL are characterized in terms of trait types, allowing QTL for similar phenotypes originally described with different terms to be linked by a single category. The markers can be linked to external databases such as NCBI via STS accessions. This will allow eventual direct browsing of genome annotations within QTL regions using Gbrowse.

As can be seen from the Entity-Relationship (ER) diagram (Fig. 4), links to genome data can be provided by adding a relationship linking markers to their genome coordinates in the Gbrowse database.

The ER diagram consists of these entities:

• QTL information, including all the details of each QTL

• Marker information, including all the details of each marker

• References, including the complete list of references

• Trait information, including the details of the traits of the cattle

• Category information, which gives details about the categories into which traits are divided

• Chromosome information, which has the length of each of chromosome measured in linkage units (Morgans).

The database is modeled such that each trait can fall under multiple categories. In addition, each QTL and marker record can have more than one reference. Finally, the QTL information and marker information are not linked directly. These data are linked dynamically by the QTL Viewer based on the position of the marker and the position of the QTL on the chromosome. This provides flexibility when the database is updated, allowing marker information and QTL information to be updated independently of each other.

Data can be added or edited using a set of web based tools that simplify database administration. Database administrators can add single QTL or easily modify QTL attributes without resorting to the command line. This makes updating the database possible by less skilled individuals such as students or technicians who may not have the training to mine the data out of the literature or have admin access to MySQL. Security settings are such that it is possible to assign users limited admin privileges over their own data, enabling them to submit and edit data but not delete QTL entries. User submitted data can be kept private and not accessible to other users until a user is ready to divulge the data for public access. This feature has been implemented to allow users to directly submit data prior to publication, allowing them to view their data in the context of public data in a genomic context.

## Utility and discussion

By ensuring the QTL records and the map information are not linked directly we make it easy to migrate the viewer to additional maps (integrated maps with arbitrary map units or RH maps) that also include map information for the QTL markers of interest. This can provide genomic visualization free from distortions caused by recombination hotspots (in the case of linkage maps) but more importantly allow QTL regions to be viewed on the best possible map, not just the map the QTL were identified from.

The QTL literature is constantly being added to, so a strategy to keep the QTL database is a necessity. At present we have settled on manual, periodic literature searches to identify new publications that we can mine for QTL data. It would be advantageous if journals that publish significant numbers of bovine QTL studies could be persuaded to require submission to our database a requirement as is done for GenBank. We hope that as our site becomes more widely used, that journals will require or at least strongly encourage authors to submit their QTL data directly to us.

## Conclusion

While there are a number of livestock genomics web sites, there is only one other site that incorporates a dynamic, genome wide display of QTL data [7]. We have concentrated on bovine QTL, but there is no reason why this viewer/database cannot be easily altered to accommodate genomes from other species with QTL data. The honey bee community has plans to adopt our database and viewer for their needs (C. Elsik, personal communication). For the bovine (or any other livestock species) genome sequence to be fully and efficiently exploited by animal scientists and biomedical researchers, map based genome coordinates must be converted from Morgans and Rays to base pairs. Therefore, as soon as the final bovine assembly is released, we expect to link the existing linkage markers to the bovine sequence using STS coordinates of the USDA markers from the map, and display the resulting QTL regions using Gbrowse. In this fashion livestock genomics researchers should be able to dispense with lengthy positional cloning exercises associated with QTL mapping. This should significantly speed up the identification and testing of candidate genes.

## Availability and requirements

Hardware requirement, any UNIX/LINUX/OSX server. Software required includes Apache, MySQL and PHP. The database and viewer code are available upon request.

## Authors' contributions

P. Polineni: designed the user interface, MySQL database and wrote the PHP code for the alpha and beta versions. Entered the original data set and contributed to the writing of the manuscript draft.

P. Aragonda: implemented refinements to the database and designed and wrote the web based data entry interface for admin users. She also entered a significant amount of the data.

S.R. Xavier: performed system administration duties, made sure database and viewer code was ported from the Linux development machine to the production machine and implemented refinements to the PHP code.

R. Furuta: contributed significantly to user interface design.

D.L. Adelson: Conceived the project, identified the data (literature/web sites), designed the data mining procedures, trained readers who mined data, contributed to interface design and database schema. Principal author of the manuscript.
